# DNA-Methylation: Master or Slave of Neural Fate Decisions?

**DOI:** 10.3389/fnins.2018.00005

**Published:** 2018-02-01

**Authors:** Stefan H. Stricker, Magdalena Götz

**Affiliations:** ^1^MCN Junior Research Group, Munich Center for Neurosciences, Ludwig-Maximilian-Universität, Munich, Germany; ^2^Physiological Genomics, BioMedical Center, Munich, Germany; ^3^German Research Center for Environmental Health, Ingolstädter Landstrasse 1, Germany and Biomedical Center, Institute of Stem Cell Research, Helmholtz Zentrum, Ludwig-Maximilian-Universität, Munich, Germany; ^4^German Excellence Cluster of Systems Neurology, Munich, Germany

**Keywords:** DNA methylation, neurogenesis, DNA modification, epigenomics, epigenetics

## Abstract

The pristine formation of complex organs depends on sharp temporal and spatial control of gene expression. Therefore, epigenetic mechanisms have been frequently attributed a central role in controlling cell fate determination. A prime example for this is the first discovered and still most studied epigenetic mark, DNA methylation, and the development of the most complex mammalian organ, the brain. Recently, the field of epigenetics has advanced significantly: new DNA modifications were discovered, epigenomic profiling became widely accessible, and methods for targeted epigenomic manipulation have been developed. Thus, it is time to challenge established models of epigenetic gene regulation. Here, we review the current state of knowledge about DNA modifications, their epigenomic distribution, and their regulatory role. We will summarize the evidence suggesting they possess crucial roles in neurogenesis and discuss whether this likely includes lineage choice regulation or rather effects on differentiation. Finally, we will attempt an outlook on how questions, which remain unresolved, could be answered soon.

## DNA methylation and other forms of DNA modifications

In 1948, Rollin Hotchkiss used paper chromatography to separate and quantify the components of DNA. To his surprise he detected not only the four nucleo-bases thymine, adenine, cytosine, and guanine, but also a “minor constituent designated epicytosine [with] a migration rate somewhat greater than that of cytosine” (Hotchkiss, 1948). As Hotchkiss had already suspected, epicytosine turned out to be a methylated form of cytosine. Thus, the first description of an epigenomic mark occurred only few years after DNA has been identified as the carrier of genetic information (Avery et al., 1944) and years before its structure has been resolved (Watson and Crick, 1953). Coincidently to these biochemical insights, first conceptual ideas arose attempting, to explain, how a single set of genetic information could give rise to the pleiotropy of cellular phenotypes (Waddington, 1957). From these early days on, epigenomic marks and epigenetic phenotypes have been closely intertwined, which lead to great discoveries but also to misconceptions, such as the perception, these two terms, *epigenetic* (“heritable traits that have their origin not in the DNA sequence”) and *epigenomic* (“reversible marks, modifications and features of DNA-implicated in epigenetic traits”) would be equivalent.

Today we know that many more DNA modifications exist. Additionally to the mark usually meant by the phrase “DNA methylation” [the methylation of cytosine at position C5 (C5-methylcytosine, 5mC)], the same base can also occur methylated on other positions [e.g., N3-methylcytosine (3mC)]. 3mC is, however, thought to represent rather a product of DNA damage than a bona fide information carrier (Sadakierska-Chudy et al., 2015). But not only cytosine can be targeted by methylation, also adenine [N6-methyladenine, (6mA); (Wu et al., 2016)]. On top, new DNA modifications on the position C5 have been discovered recently, which are generated by DNA demethylation pathways (Booth et al., 2015, Figure 1). The first of these 5mC oxidation products to be reported was 5hmC (C5-hydroxymethylcytosine) (Kriaucionis and Heintz, 2009; Tahiliani et al., 2009); 5fC (C5-formylcytosine), and 5caC (C5-carboxylcytosine) followed later. Although 5hmC has been described to occur in animal tissues (e.g., mouse brains) already in the 70s (Penn et al., 1972), its relevance was not recognized as it was widely interpreted as a product of DNA damage (Privat and Sowers, 1996). Today we know that 5hmC and 5caC are not necessarily transient marks occurring solely in a sequence of chemical reactions; instead they can appear quite stable at least under some circumstances (Bachman et al., 2014, 2015).

**Figure 1:**
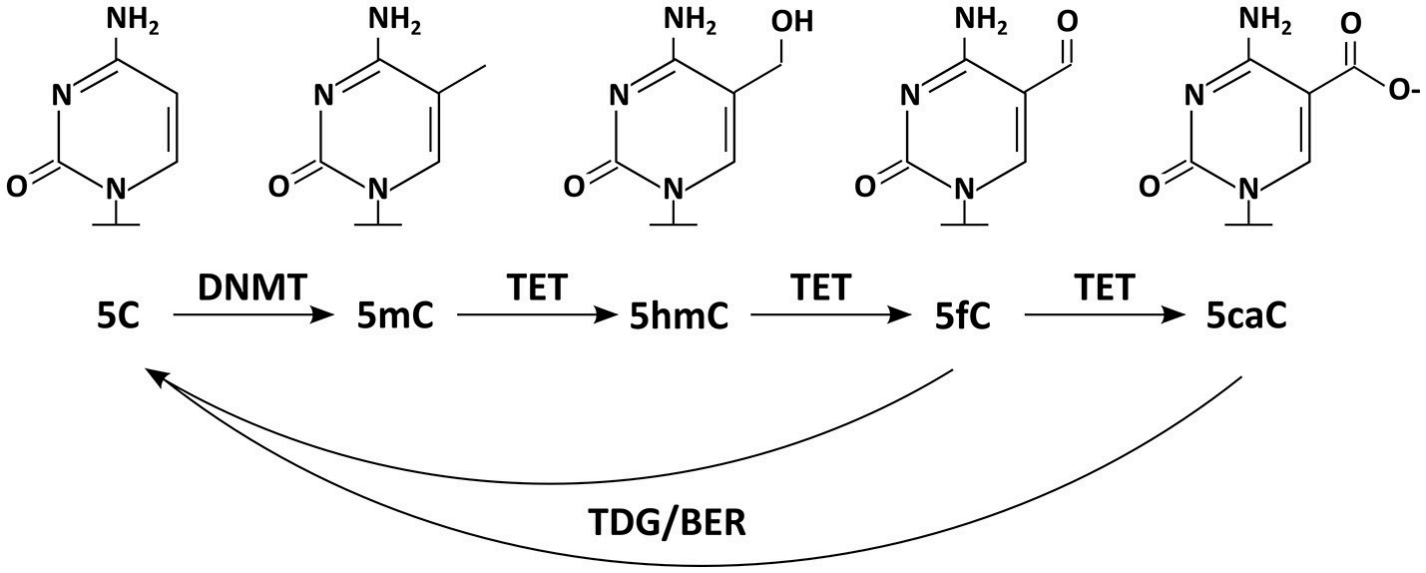
Chemical structures of DNA modifications: five DNA modifications and relevant enzymes are depicted. DNMTs methylate 5C resulting in 5mC, which can be further modified by TET enzymes to 5hmC, 5fC, and 5caC. Enzymes of the TDG/BER pathway have been implicated in removal of the DNA modifications.

In the following, we will give a short overview about the distribution of DNA modifications and discuss how they are established. We will then present the suggested roles for DNA modifications in gene expression control and review how those have been implicated into regulating lineage decisions during brain development. We finish with re-evaluating the scientific evidence for DNA methylation marks controlling neurogenesis and discuss recent technical advances to study their function at precise sites in the genome. Although we mention several biological processes and all known DNA modifications in this review, we will focus on the role C5-methylcytosine plays in neurogenesis and neuronal maturation.

## Epigenomic distributions of DNA modifications

Although DNA modifications are common in bacteria (e.g., m6A, N6-methyladenine; m5C, C5-methylcytosine; m4C, C4-methylcytosine) (Chen et al., 2014), many eukaryotic model systems have no or only traces of 5C methylation. Neither *Saccharomyces cerevisiae* nor *Caenorhabditis elegans* possess this epigenomic mark (Shin et al., 2014). In *Drosophila melanogaster* it is very rare and has only lately been confirmed (Lyko et al., 2000). This remarkable absence of canonical DNA methylation in the three most frequently used genetic model systems might be one reason its universal significance for gene expression and cellular phenotypes is still not known. Consequently, today, 70 years after its discovery, the discussion about how frequent *functional* DNA methylation marks are, is still ongoing (Stricker et al., 2017). In this context it should be mentioned, however, that the genomes of *Caenorhabditis elegans* and *Drosophila melanogaster* have recently shown to possess significant levels of m6A (Greer et al., 2015; Zhang et al., 2015).

**Figure 2:**
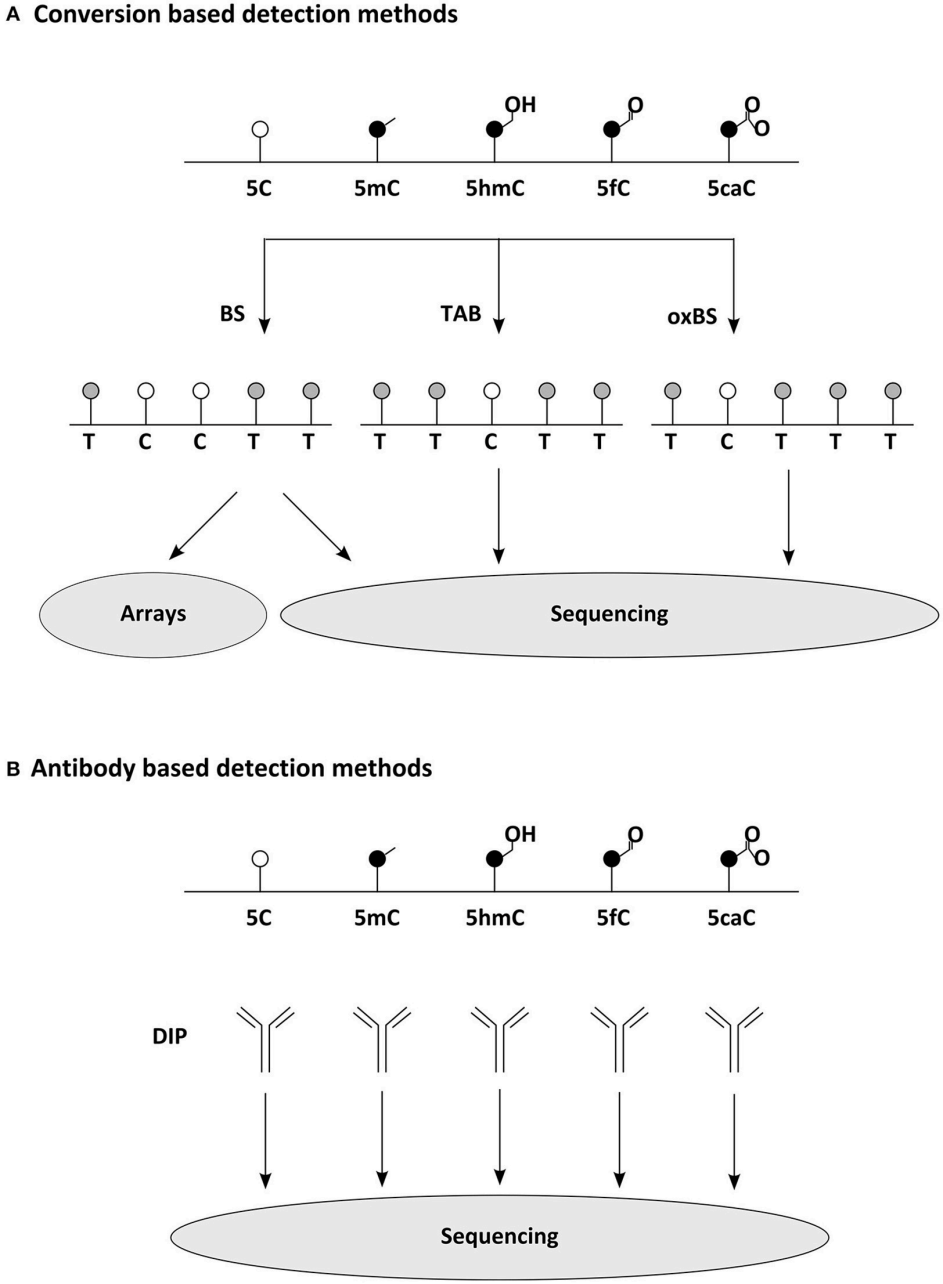
Common methods for widespread detection of DNA modifications. **(A)** Conversion based detection methods. Bisulfite (BS) sequencing, oxidative bisulfite (oxBS) sequencing, and Tet assisted BS (TAB) sequencing enable the epigenomic distinction of 5C, 5mC, and 5hC, while similar techniques separating 5fC and 5caC have been developed as well (Plongthongkum et al., 2014). Sequence below indicates readout expected in NGS. For comprehensive analysis of DNA modifications several detection methods must be combined. **(B)** Antibody based detection methods. DNA Immunoprecipitations (DIP) using modification specific antibodies allow the quantitative analysis of epigenomic distribution (making use of NGS or arrays). meDIP (methylated DNA immunoprecipitation) has been the archetype of this methodology (Weber et al., 2005), but several variants for other DNA modifications have been reported as well recently (comprehensively reviewed in Plongthongkum et al., 2014).

In mammalian cells m5C is rather frequent it occurs mainly in pairs of CpGs, in which between 80 and 90% of cytosines are methylated (Hon et al., 2013). Interestingly, those 10–20% CpGs found to be unmethylated are not distributed randomly throughout the genome, but concentrate on so called CpG islands, which mostly coincide with gene promoters. Indeed, around half of mammalian transcripts begin in a CpG island (Bird, 2002). Until recently, it was believed that 5mC occurs in mammalian cells exclusively in the CpG context. That this is not necessarily the case has been shown with the help of new methods for epigenomic analysis of DNA modifications (Figure 2): first, in human pluripotent stem cells, in which 25% of m5C occurs at CpH sites (with H = A, C, or T) (Lister et al., 2009), later this has been found also in (mouse and human) brain samples. Other tested somatic cells are, as far as we know, mostly devoid of such non-CpG methylation as far as we know (Xie et al., 2012; Lister et al., 2013). Similarly to non-CpG methylation, C5-hydroxymethylcytosine has also been first described in DNA derived from pluripotent and brain cells (Kriaucionis and Heintz, 2009; Tahiliani et al., 2009). Especially hypothalamus, cerebral cortex, and hippocampus have been reported to be rich sources of hm5C (Munzel et al., 2010), occurring almost at the rate of one sixth of m5C (Shin et al., 2014), often on enhancers (Yu et al., 2012). N6-methyladenine was found in mouse ES cells, in which it occurs particularly on young LINE elements; they themselves are enriched on the X-chromosome (Wu et al., 2016).

## Establishment and removal of DNA modifications

DNA methylation is catalyzed by a group of enzymes, the DNA methyltransferases, which catalyze the transfer of a methylation residue from S-adenosyl-L-methionine to C5 of cytosine. In mammals these consist of Dnmt3a and Dnmt3b, the *de novo* methyltransferases and Dnmt1, that maintains methylation through the cell cycle by copying CpG methylation patterns from the mother to the newly synthesized strand. Rodents have recently been shown to possess an additional *de novo* DNA methyltransferase, Dnmt3c, evolved through a gene duplication of Dnmt3b (Barau et al., 2016). The mammalian enzyme responsible for adenine methylation is currently unknown. Dnmt3a has been reported to occur in two different forms, due to alternative promoter usage. Although this is not uncommon for protein coding genes, it might be relevant for the methylome, since in cell lines Dnmt3a1 (the full length protein) and Dnmt3a2 (the short isoform) have been reported to occupy very different locations in chromatin. While Dnmt3a1 is found mainly in heterochromatin, Dnmt3a2 is associated with euchromatic regions (Chen et al., 2002). The two remaining members of the Dnmt family, 3L and 2, are paralogs, which either lost enzymatic activity or methylate RNA (Goll et al., 2006; Ooi et al., 2007). While the *de novo* enzymes Dnmt3a and Dnmt3b are necessary to set DNA methylation marks (on CpG and likely also non-CpG positions) (Guo et al., 2014), Dnmt1 ensures its long term inheritance. It is acting on hemi-methylated DNA, occurring after DNA replication (or DNA repair) and transfers a methyl group to the cytosine on the unmethylated strand. Obviously, this depends on the palindromic base composition of CpG di-nucleotides. mCpH sites lack a cytosine residue on the second DNA strand and thus, are certainly asymmetrically inherited to the progeny of pluripotent and neural stem cells. Whether this has however, any functional consequence has remains to be shown.

That Dnmt1 constantly antagonizes passive DNA demethylation is widely accepted. Whether there are any active processes selectively removing DNA methylation marks from certain epigenomic locations has been a controversial issue for a long time. Over the last decades there have been a series of reported findings of DNA demethylases (wittily summarized by Ooi and Bestor, 2008). In contrast to those, recent candidates have been received more favorably (Wu and Zhang, 2010). Today it is widely accepted, that a number of enzymes contribute on the de-methylation of 5mC. First of all the members of the ten-eleven translocation family of enzymes (Tet1, Tet2 and Tet3) oxidize 5mC to 5hmC. But Tet activity does not necessarily stop at this point, as these enzymes can further oxidize 5hmC to 5fC and subsequently to 5caC (Figure 1) (He et al., 2011; Ito et al., 2011). These marks are then thought to be lost passively or removed by the thymine DNA glycosylase (TDG), a forerunner of the base excision repair (BER) (Yu et al., 2012; Zhang et al., 2012). Also other proteins and enzymes involved in DNA repair (e.g., GADD45/AID/APOBEC) have frequently been implicated in active DNA de-methylation (Rai et al., 2008; Bhutani et al., 2010, 2011), although their contributions to global methylomic changes are still being discussed (Nabel et al., 2012).

## General concepts for potential functions of DNA modifications

DNA methylation has been implied in regulation of gene transcription already in the late 60s (Harrisson, 1971; Scarano, 1971; Holliday and Pugh, 1975; Riggs, 1975) and often still is; although it has become clear that it likely plays a much less general role than believed originally. But why has DNA methylation become the one epigenomic mark most frequently connected to epigenetic gene silencing in the first place? There are plenty of answers to this question, which we are neither able to discuss fairly, nor to list comprehensively; we think however, that most of the concepts and experimental evidence gained during the decades can be grouped into four types, which we will address below. First, the biochemical features of DNA methylation, its life cycle and inheritance make it a prime candidate for a developmental epigenetic mark; second, global correlations between the presence of DNA methylation and the activity state of DNA in the nucleus do occur; third, DNA methylation is necessary for normal animal development and finally, on some individual model loci a functional effect of DNA methylation on restricting transcription is clearly evident. Hereafter, we will discuss the evidence for the above criteria in establishing the previous model, namely a role of DNA-methylation in repressing alternative fates. Subsequently we will proceed to discuss experimental evidence testing this model. Data from pluripotent stem cell differentiation and mouse models *in vivo* (section Mouse Models) demonstrate that no fate switch to an alternative fate occurs even when most or all of methylation marks have gone (see section Mouse Models). Conversely, phenotypes appear late in brain development, often at postnatal stages, indicating rather that maturation processes are affected (Tables 1, 2).

**Table 1:** Published knockout mouse models and their reported phenotype during brain development and in the adult brain.

**Gene**	**Type of Mutant**	**Cells/Time**	**Phenotype**	**References**
Dnmt1	Nestin-Cre	NPCs/E12	Premature glial marker induction, neuron loss.	Fan et al., 2005
	CamK-Cre	Neurons	No obvious effect.	Fan et al., 2001
	Nestin-CreER^T2^	NPC/adult	Decreased survival of hippocampal neurons.	Noguchi et al., 2015
	CamK2a-Cre93	Excitatory neurons in the mouse forebrain	Deficits in learning and memory (+Dnmt3a).	Feng et al., 2010
	Emx1-Cre	Early cerebral cortex	Cortical degeneration, neuronal loss.	Hutnick et al., 2009
	Olig1-Cre	Early OPC progenitors	Oligodendrocyte Maturation defect, ER Stress.	Moyon et al., 2016
	Chx10-Cre	Retinal NSCs	Defective photoreceptor differentiation.	Rhee et al., 2012
	Rx-Cre	Early retina anlage	Photoreceptor degeneration (+Dnmt3a,b).	Singh et al., 2017
Dnmt3a	Nestin-Cre	NPCs/E9-E10	Motor neuron loss.	Nguyen et al., 2007
	Full K.O.		Impaired postnatal differentiation, repression of neurogenic genes.	Okano et al., 1999; Wu et al., 2010
	CamK2a-Cre93	Excitatory neurons in the mouse forebrain	Deficits in learning and memory (+Dnmt1).	Feng et al., 2010
	Plp-CreER(t)	Adult OPCs	Remyelination impaired.	Moyon et al., 2017
Dnmt3b	Full K.O.	E11.5	Rostral neural tube defects.	Okano et al., 1999
Uhrf1	Emx1-Cre	E10–E12	Postnatal neurodegeneration, IAP activation.	Ramesh et al., 2016
Tet1	Full K.O.		Impaired adult hippocampal neurogenesis, Activity induced gene activation affected, Memory formation and extinction affected, When outbred, embryonic lethal (forebrain defects).	Rudenko et al., 2013; Zhang et al., 2013; Khoueiry et al., 2017
MBD1	Full K.O.		Reduced adult hippocampal neurogenesis, Expression of endogenous viruses, Aneuploidy, Impaired LTP in DG.	Zhao et al., 2003
MBD2	Full K.O.		Maternal behavior affected in adult mothers.	Hendrich et al., 2001
MeCP2	Full K.O.		Impaired neuronal maturation in Hippocampus.	Smrt et al., 2007
GADD45b	Full K.O.		Reduced activity induced proliferation of progenitor cells in the hippocampus.	Ma et al., 2009

**Table 2:** Predictions and experimental support of two models for main function of DNA-methylation in neurogenesis.

**Predictions model 1**	**Met (+)/unmet (-)**	**Predictions model 2**	**Met/unmet**
Early phenotype	–	Late (postnatal) phenotype	+
Appearance of alternative fate	–	Maintenance of immature hallmarks	+
mRNA up-regulation of alternative cell fate genes	−(except GFAP)	Failure to down-regulate progenitor-specific mRNas	+

*Model 1: DNA-methylation represses alternative fates vs. Model 2: DNA-methylation represses immature hallmarks to allow full maturation*.

### The life cycle of DNA methylation levels and its inheritance

Since decades it is relatively undisputed that mammalian development has to provide a molecular memory restricting the options of each individual cell to express or adopt cell identities. Until recently, cellular potency was believed to be a one way street, with continuously less choices as development progresses. This has been put in a nutshell by the iconic depiction of the epigenetic landscape conceived by Waddington (1957). Although we know today, that we can revert development (Takahashi and Yamanaka, 2016) or provide direct shortcuts (Masserdotti et al., 2016), the basic question remains: What informs and restricts cellular identity during development? Very early on DNA methylation has been considered to be the prime candidate fulfilling this role. The reason for this has much to do with the dynamics of the mark itself as m5C is a quite stable modification. Many m5C marks are set early in development (some even in the germ line, e.g., the imprints), but can often still be found in somatic cells. This stability is mainly provided by Dnmt1, which faithfully copies the methylation signature from the mother strand after each round of DNA replication. Despite its heritability over cell divisions, DNA methylomes also undergo significant changes during development, both globally and locally. A good example for global methylation changes is occurring during early embryogenesis. Sperm and oocyte each show high overall methylation levels. During the first cell divisions of the zygote, DNA methylation gets remodeled. Both, the maternal and the paternal epigenome get de-methylated, interestingly, however, with very different dynamics. While the paternal genome is immediately actively demethylated, the maternal genome undergoes passive DNA demethylation during continuous DNA replications (Messerschmidt et al., 2014). Thereafter, rapid re-methylation occurs on both genomes with the blastocyst stage, coincidently at the time cells loose totipotency and specify (Reik et al., 2001). Even though such dramatic changes are not recurring later in development; there are plenty local DNA methylation changes occurring in each cellular lineage, resulting in rather specific methylomes (Bernstein et al., 2007), which can not only be used to predict cell type, but even age (Horvath, 2013).

### Correlations between DNA methylation and transcription

Early on it has been noticed that some DNA methylation changes occurring during development can correlate to transcriptional changes. The most impressive example, maybe because of its scale, is the hypermethylation on CpG island promoters found on inactivated X-chromosomes in female mammalian cells (Lock et al., 1986; Singer-Sam et al., 1990), while the genetically identical copies on the active X-chromosome remain unmethylated. But also promoters of lineage specific genes, like MyoD or various globins, being studied since decades in primary and immortalized cells, have been found to attract DNA methylation when the respective genes get downregulated (Jones et al., 1990). More recently, these concepts have been refined, as it has been reported that those DNA methylation changes occurring during development and correlating to transcriptional differences among tissue types, do rarely involve entire CpG islands, but more often their mere borders (Irizarry et al., 2009). It should, however be mentioned, that most methylated sites in the genome lack predictive value and quite some methylated loci correlate rather to active transcription than gene silencing (Niesen et al., 2005; Irizarry et al., 2009; Bahar Halpern et al., 2014; Zhu et al., 2016).

### Genetic manipulation of DNA modifications

Further hints into the functional relevance of DNA modifications were given by the generation of genetically modified mouse lines lacking parts of the machinery necessary for their deposition or removal. Thus, it has been shown that the ability to set and propagate DNA methylation marks is absolutely essential to undergo normal embryonic development, since animals lacking the *de novo* DNA methyltransferases Dnmt3a and b or the maintenance methyltransferase Dnmt1 are not viable (Li et al., 1992; Okano et al., 1999). In contrast to this, the consequences of losing members of the Tet family of enzymes seem less severe. ESCs and mice lacking Tet1 [showing a considerable loss of 5hmC (~20–40%)] are overall viable and only few genes are significantly mis-regulated (Dawlaty et al., 2011), although it has been reported that in non-inbred mice Tet1 is essential for embryogenesis (Khoueiry et al., 2017). A combined loss of Tet1 and 2 lead to a larger number of intermittent phenotypes, but mice lacking both proteins can be born viable and fertile (Dawlaty et al., 2013). Only when all three Tet proteins are depleted differentiation of pluripotent cells is largely impaired possibly due to dysregulation of important developmental genes (Dawlaty et al., 2014). Also depletion of the TDG affects animal development and accumulation of erroneous DNA methylation marks which is compatible with its suggested role in the DNA de-methylation pathway. However, reported changes are comparatively moderate and involve mostly genes known to swiftly attract DNA methylation, like the Hox genes (Cortazar et al., 2011).

### Model systems of DNA methylation function

Several model systems have over the years suggested a direct role for DNA methylation in transcriptional regulation. An early example is the *in vitro* methylation of DNA which has been shown to prevent transcription of exogenous copies of globin genes when transfected into mammalian cells (Busslinger et al., 1983). But also the discovery of genomic imprinting, a phenomenon of parental specific gene expression in the embryonic or adult offspring (Barlow et al., 1991; Bartolomei et al., 1991; Ferguson-Smith et al., 1991), delivered much needed evidence. It has been found that loci containing genes with imprinted expression contain a differentially methylated region, established through differences in gametic methylation patterns, which serve as imprinting control regions (ICEs). Genetic approaches resulting in loss of ICEs, imprinted DMRs or global DNA methylation eliminate parental specific gene expression, strongly suggesting a direct functional role for these DNA methylation marks in imprinted gene regulation (summarized in Barlow and Bartolomei, 2014).

## Theoretical models of transcriptional regulation by DNA modifications

**Figure 3:**
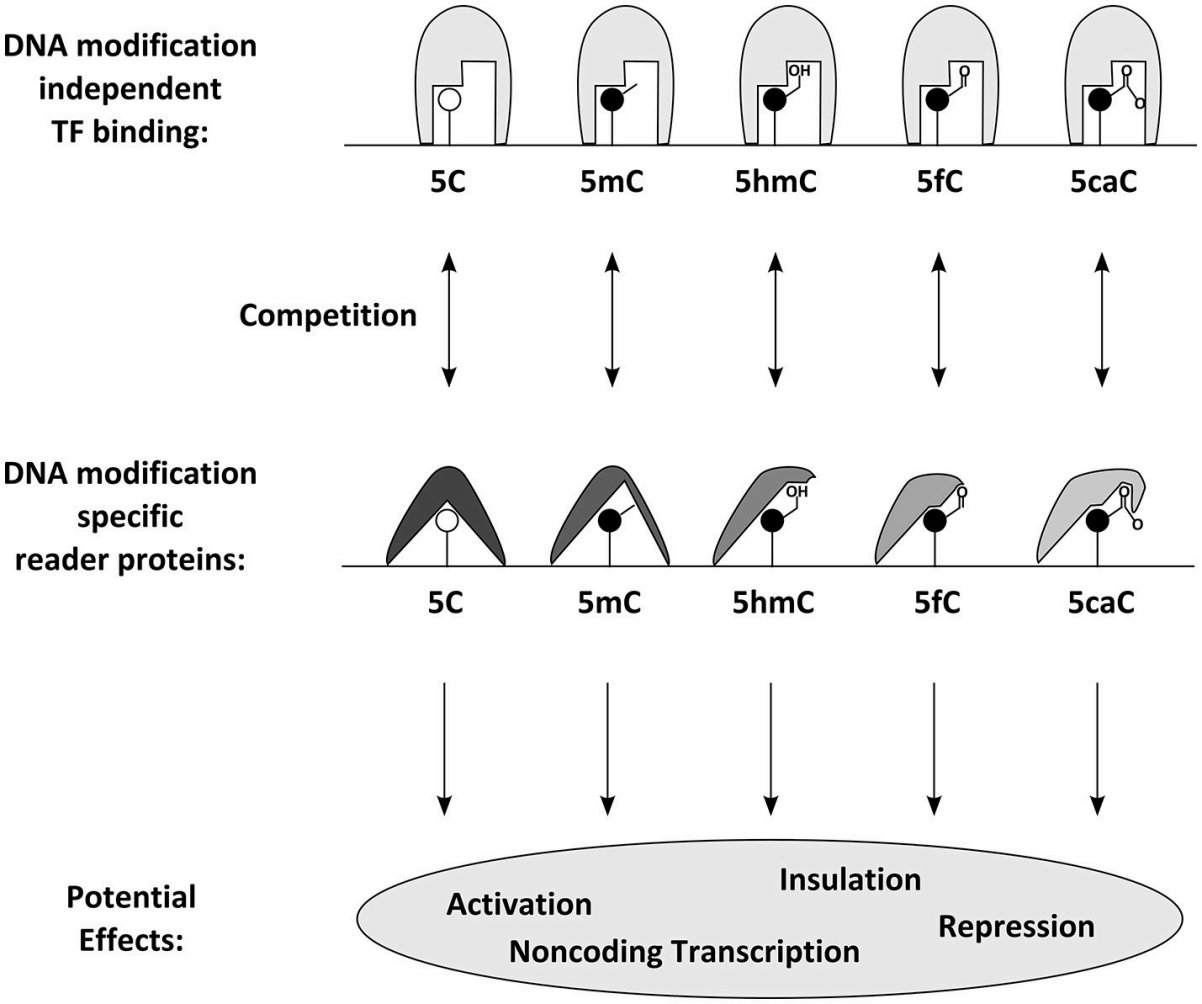
Proposed molecular effects and consequences of DNA modifications: DNA modifications can be specifically bound by reader proteins. Those can either have a direct effect or compete with DNA modification independent transcription factors and thus influence transcription through gene activation, repression, non-coding transcription or insulation.

How DNA methylation influences transcription was long elusive. The mechanisms by which DNA methylation of ICEs regulate imprinted gene expression vary and span from controlling expression of long non-coding RNAs (Lyle et al., 2000; Seidl et al., 2006) to interfering with the binding of the common chromatin protein CTCF on insulator elements (Bell and Felsenfeld, 2000). The most popular model for the effect of DNA methylation entails (consistent with active genes containing many 5mC residues in their bodies) that DNA methylation is not directly interfering with transcription. One common assumption is that it is rather the DNA binding affinity of transcription factors which is influenced by DNA methylation (Tate and Bird, 1993; Zhu et al., 2016). While many transcription factors are thought to be impaired by DNA methylation, some special transcription factors bind methylated DNA specifically (Figure 3). In this model, the group of proteins involved in gene regulation by DNA methylation can be divided in writer (e.g., the aforementioned DNMTs), eraser (e.g., TET proteins), and reader proteins. The latter can sense the presence of DNA methylation marks and respond with altered DNA binding affinity. Characterization of methyl binding proteins was a tedious task that is still ongoing today. Firstly discovered was a family of transcription factors defined by the possession of a protein domain, shown to prevalently bind to 5mC containing DNA *in vitro*. This so called MBD (methyl CpG binding domain) family of transcription factors has five known members (MBD1, MBD2, MBD3, MBD4, and MeCP2) (Hendrich and Bird, 1998; Zhu et al., 2016). Recent technological development has enabled the genome-wide characterization of their DNA binding features and elucidated methylation dependent and (particularly in the case of MBD3) independent DNA binding (Baubec et al., 2013). Complementary approaches helped discovering a large series of new candidate proteins that *in vitro* bind at least some of their possible binding motives specifically in the methylated form (comprehensively reviewed in Zhu et al., 2016), including many classical transcription factors like the pioneering factors Klf4 (Hu et al., 2013) and Kaiso (Prokhortchouk et al., 2001). A specialty among known methylation binding transcription factors is Uhrf1, a critical partner of Dnmt1, as it has been shown to recognize *hemi-methylated* DNA in its binding motive (Fang et al., 2016). Recent approaches aiming to discover reader proteins also for other DNA modifications. These efforts resulted in candidate lists for 5hmC- (e.g., Uhrf1 and Uhrf2), 5fC- (e.g., members of the NuRD complex), and 5caC-binding proteins (Frauer et al., 2011; Iurlaro et al., 2013; Spruijt et al., 2013) and indicated that MeCP2 is binding 5mC in both, CpG and CpH sites as well as other cytosine modifications (Mellen et al., 2012; Guo et al., 2014; Gabel et al., 2015). While much needs to be learnt about the *in vivo* roles of these reader proteins and few have been investigated in the context of neurogenesis and DNA methylation so far, recent analysis of Uhrf1 supports its key role in DNA-methylation homeostasis during development and reveals key requirements for later neuronal differentiation processes (Ramesh et al., 2016).

### Arguments against global roles

Classical epigenetic research on model loci has provided functional examples and mechanistic models; through epigenomic approaches we can acknowledge how complex and dynamic epigenomes present themselves. Thus, to date the most pressing question in epigenetics is not so much, whether chromatin models of epigenetic gene regulation are correct, but rather how ubiquitous their functional relevance is; it is, for example, completely unclear, how many genes (and phenotypes) are significantly regulated by DNA modifications during development and disease. This is especially relevant for DNA methylation, which is rather frequent throughout the genome and has been extensively mapped. Interestingly, however, quite some data argues against the idea that the aforementioned models could be easily translated to any locus or transcriptional unit.

One of the earliest arguments against a ubiquitous role for DNA methylation in gene regulation was the finding that most epigenetically silenced promoters do not appear heavily methylated during development and, related to this, that those that do, often gain DNA methylation after gene expression is lost (Bird, 2002). But there is not only evidence that developmental gene silencing does not depend on DNA methylation changes, recent approaches using cancer tissue derived induced pluripotent stem cells suggest also that removal of disease associated DNA methylation marks does not influence tumorigenicity of cancer cells significantly (Stricker et al., 2013; Chao et al., 2017). The most convincing argument might, however, come from genetically engineered embryonic stem cells lacking all six active copies of DNA methyltransferases [Dnmt1, Dnmt3a and Dnmt3b, Dnmt3c is not expressed in embryonic stem cells (Barau et al., 2016)]. These triple knockout ESCs have undetectable levels of DNA methylation in their genome. Surprisingly these cells are not only viable and macroscopically normal; they also possess very few mis-regulated genes. Moreover, as subsequently revealed by DNAse hypersensitive site analysis, very few transcription factors change their binding spectrum once DNA methylation is lost in these cells (Domcke et al., 2015). Similarly unexpected is the finding, that the complete loss of TET proteins in differentiating ESCs only results in a moderate increase of 5mC (Dawlaty et al., 2014). These and other findings suggest that our current models of epigenetic gene regulation might be incomplete and have to be revisited in order to elucidate the function of DNA modifications.

## DNA methylation in neurogenesis

**Figure 4:**
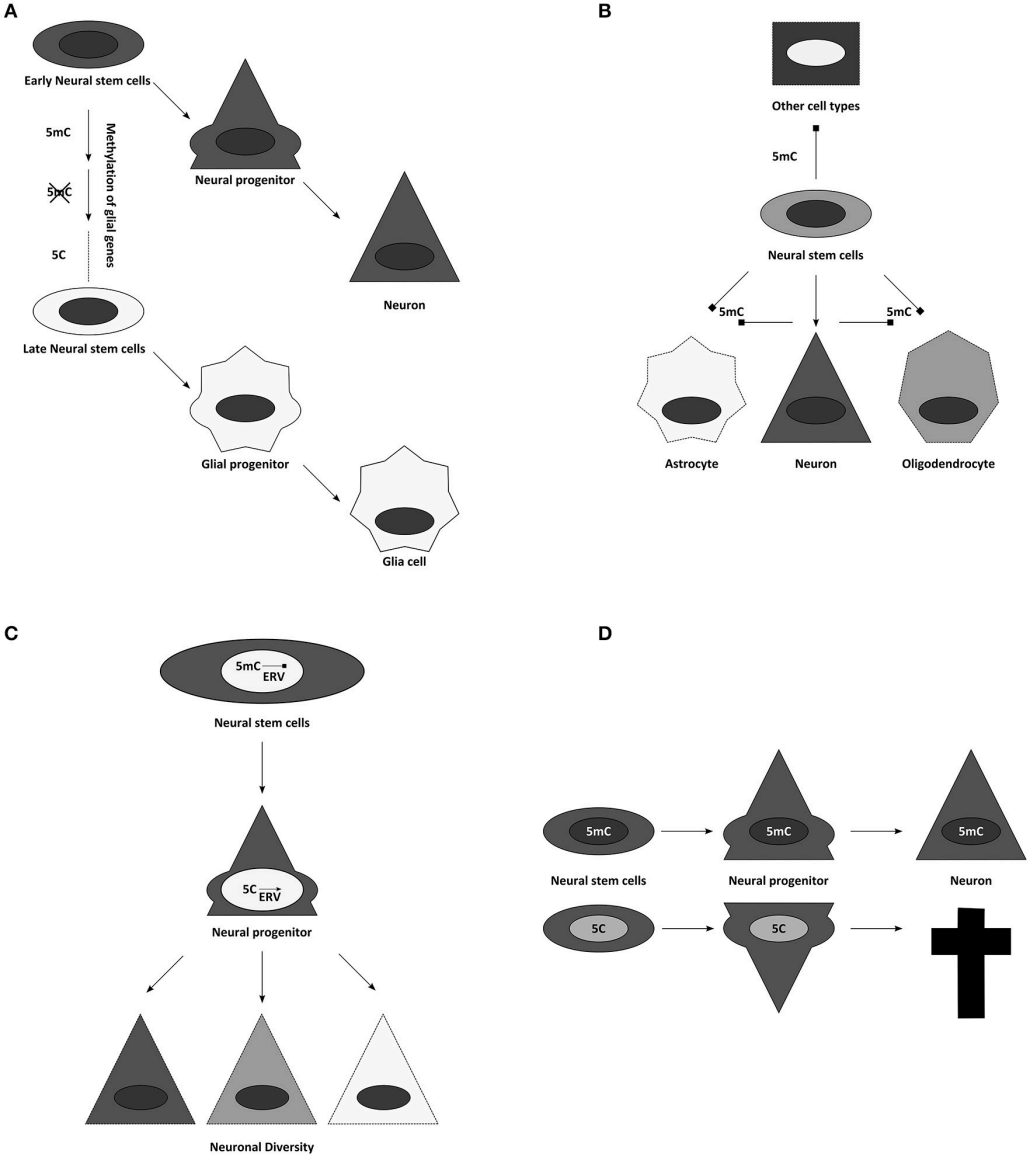
Suggested influences of DNA methylation on neurogenesis. **(A)** Temporal progression of DNA methylomes might influence the potential of neural stem and progenitor cells. **(B)** Cell specific methylomes, here 5mC for simplification, might be responsible for neural cell identities. They could not only influence lineage choices, but might also simultaneously block alternative fates. **(C)** Through controlling activity of transposon derived sequences, DNA methylation has been implicated in contributing to neuronal diversity. **(D)** Global alterations of DNA modifications often result in cell death during differentiation.

The above data prompt the question of how important DNA methylation would be in development. Development can be seen as a series of cellular fate restrictions and hence DNA methylation has been suspected to be involved in these processes. For example, neural stem cells (NSCs) become progressively restricted in the generation of neurons and later retain only the potential to generate glial cells in most brain regions (Figure 4A). Interestingly, the earliest restriction in fate is spatial and special to the nervous system as it is mediated by patterning and occurs in regard to the region the NSCs reside in (Kiecker and Lumsden, 2005). Even prior to the generation of neurons or glial cells, NSCs are already committed to generate region-specific subtypes, e.g., excitatory projection neurons in the cerebral cortex. The second fate restriction is temporal, with neurons of deep cortical layers generated earlier than neurons of the upper layers of the mammalian neocortex (Molyneaux et al., 2007). Indeed, transplantation experiments revealed that early NSCs have the potential to generate neurons of all cortex layers while late NSCs loose the potential to generate deep layer neurons (Frantz and McConnell, 1996), suggesting progressive fate restriction in regard to neuronal subtype specification. As recently confirmed with new tools for clonal analysis (Gao et al., 2014), this occurs via an asymmetric mode of division, by which a NSC generates sequentially different neuronal subtypes sometimes directly and sometimes via intermediate, transit-amplifying progenitor cells. Only after generating all these neurons, the NSC eventually switches to generate glial cells. Thus, the developmental time point predicts whether the stem cell progeny commits to a neuronal or a glial fate (Götz et al., 2016). Not well-understood is, however, how the sequential fate specification is achieved, how the previous fates are repressed and how new lineages are installed.

DNA modifications have been attributed diverse roles in this process. For example, it has been suggested that gliogenesis occurs late, because glial genes are repressed during most of neurogenesis by DNA methylation (Takizawa et al., 2001). This finding could be expanded to the concept that cellular methylomes define cell identities directly (Figure 4B). A certain combination of DNA methylation marks might safeguard the faithful expression of adequate cellular programs, while simultaneously repressing inappropriate transcriptional networks (Figure 4B) (Lee et al., 2014). Accordingly, temporal changes in DNA methylation may then also allow the sequence of neuronal fates generated during development (Figure 4A) (Takizawa et al., 2001; Sanosaka et al., 2009). In agreement with this is the recent finding that human GABAergic interneurons and glutamatergic projection neurons indeed differ vastly in their distribution of DNA modifications (Kozlenkov et al., 2016). This concept of DNA-methylation fixing fates and repressing alternatives predicts ectopic fates to be generated upon interference with DNMTs or TETs, and we will see below that evidence from mouse mutants does not support this prediction.

But first we will consider another important role for DNA methylation, where its repressive role is clearly evident, namely repressing endogenous retroviral elements (ERVs, Groh and Schotta, 2017). In all mammalian cells, the highest proportion of DNA methylation is found on repetitive regions, representing transposons, retrotransposons, or sequences derived from these (Crichton et al., 2014). Therefore, it has been frequently suggested that the main function of DNA methylation might be to silence these intragenomic parasites (Yoder et al., 1997). However, these elements might also have important roles during neurogenesis (Figure 4C). On one hand active transposition could contribute (Erwin et al., 2014), on the other many regulatory elements in the genome are evolved from or influenced by endogenous retroviruses (ERVs) domesticated for gene expression (Rebollo et al., 2012; Fasching et al., 2015). Thus, it is not unlikely that genome protective and gene-regulatory roles of DNA methylation follow similar principles. It has been suggested that ERVs contribute to the enormous neuronal diversity and plasticity of the neuronal lineage (Rebollo et al., 2012; Erwin et al., 2014). Epigenetic mechanisms control ERV activity and thus regulate local chromatin remodeling, transcription and potentially their translocation (Figure 4C). This would imply an important evolutional role to the pronounced increase of viral elements in the genome during mammalian phylogeny. However, there are only few experimental options to unequivocally assess the function of DNA modifications during cortical development and thus to strengthen these hypotheses, including: the characterization of the availability of the enzymatic machinery during development; the epigenomic analysis of DNA modifications during cortical neurogenesis; and finally, the use of genetically modified mouse models, possessing altered amounts or distribution of DNA modifications.

### Expression of the DNA modifying machinery during cerebral cortex neurogenesis

The developing as well as the adult brain expresses most proteins implicated in the regulation of DNA modifications. Dnmt1 is ubiquitously present in fetal and full grown mouse brains (Goto et al., 1994); i.e., even in postmitotic neurons and glia. But also the *de novo* methyltransferases are detectable in the nervous system. Dnmt3a is prominently expressed e.g., in neural stem and progenitor cells of the ventricular and subventricular zone of the developing cerebral cortex (E10.5–E17.5), as well as in postnatal neurons and the oligodendrocyte lineage (Moyon et al., 2016), while it is mostly absent in astrocytes (Feng et al., 2005). Dnmt3b can only be detected in the SVZ early (E10.5–13.5), not later during development (E15.5) (Feng et al., 2005; Moyon et al., 2016). The newly discovered rodent Dnmt3c lacks expression in brain as far as we know (Barau et al., 2016). Neural expression of the three Tet proteins has been reported as well (Khoueiry et al., 2017), with Tet3 most dominant and Tet1 most feeble, and with little modulation between newborn and adults (Szulwach et al., 2011) or brain regions (Szwagierczak et al., 2010), but dynamic changes during oligodendrocyte differentiation (Zhao et al., 2014). Interestingly, Tet3 expression has been found to be amplified by synaptic activity in cultured hippocampal neurons (Yu et al., 2015). Additionally, many methyl binding proteins are present in the nervous system, sometimes in remarkably selective patterns. A typical example is Mbd1, expressed commonly in neurons, but not detectable in astrocytes (Zhao et al., 2003). Thus, the availability and the (at least partially) dynamic expression of the DNA methylation and de-methylation machinery during cell fate commitment and differentiation is indeed in line with potential roles for this epigenomic mark in these processes.

### Epigenomic distribution of DNA modifications during neurogenesis

First indications about the cell type specific distribution and dynamics of DNA methylation during neurogenesis (and its relation to other epigenomic marks and transcription factor binding) have been gained from differentiation of embryonic stem cells or neural progenitor cells (Meissner et al., 2008; Stadler et al., 2011). Profiling of pluripotent and neural stem cells revealed for example, that regions with low methylation show the most dynamic DNA methylation changes during development. Moreover, these are frequently overlapping with regulatory sequences of important cell fate factors (like Pax6) and are dependent on transcription factor activity in some tested cases, as DNA binding (of the neural repressor REST for example) is necessary and sufficient to evade high DNA methylation levels on its binding sites (Stadler et al., 2011).

The recent development of affordable technology for DNA methylome analysis made the investigation of human brain samples practicable as well (Figure 2). Large cohorts of human prefrontal cortex samples revealed the dynamic changes occurring during development and aging of the brain (Hernandez et al., 2011; Numata et al., 2012; Jaffe et al., 2016). These studies indicate that, although methylation differences are occurring in different scales, either at individual CpGs, at differentially methylated regions (DMRs) or at even larger domains, most changes are established during development and childhood, while methylomes are less plastic later in life. These findings likely point to differences in cellular composition rather than developmental dynamics and thus demonstrate the predicaments when heterogeneous cell populations are examined. Analysis of more homogeneous cell populations allow deeper insights, e.g., revealing how in the developing and adult frontal cortex 5mC patterns distinguish cell types (Lister et al., 2013) or that methylated CpH sites are almost absent from (NeuN negative) non-neuronal cells (Lister et al., 2013). Instead, CpH methylation is generated *de novo* during neuronal maturation both in mouse and human cells (Lister et al., 2013; Guo et al., 2014) and parallels synaptogenesis and neuronal diversity (Lister et al., 2013; Mo et al., 2015). Remarkably, studies also indicate that methylation marks occurring in regulatory regions are more indicative of transcriptional repression when falling on CpH rather than on CpG sites (Mo et al., 2015). The first characterization of 5hmC dynamics was linked to the development of reliable methods mapping this mark epigenome-wide (Figure 2). Using hMeDIP for example has shown that in contrast to 5mC, the cellular amount of 5hmC is significantly increasing when neural stem and progenitor cells are differentiating to neurons (Hahn et al., 2013). A similar developmental dynamic has also been detected during *ex vivo* analysis of mouse cortices and human brain samples (Szulwach et al., 2011; Lister et al., 2013; Wen et al., 2014; Vogel Ciernia and LaSalle, 2016). Interestingly, newly acquired 5hmC often associates with regulatory elements of neuronal genes (Szulwach et al., 2011; Wang et al., 2012) and are solely detectable at CpG sites (Lister et al., 2013). Bisulfite sequencing of DNA derived from adult mouse dentate granule neurons before and after synchronous neuronal activation *in vivo*, revealed that some DNA methylation marks do not behave as stable as commonly expected and rather suggested that around 1% of analyzed 5mC sites fulfill the criteria of activity induced de-methylation (Guo et al., 2011) with yet elusive function. Taken together profiling of DNA methylation in mammalian brain cells from both *in vitro* and *ex vivo* models indicate that diverse cell populations differ significantly in their methylome and that these changes can swiftly emerge at meaningful sites, indicating that they could contribute to shape cellular functions.

### Mouse models

Genetically modified mouse models of all known writers of the DNA methylation machinery have been generated to functionally test the global relevance of this epigenomic modification. The full knockout for the *de novo* methyltransferase Dnmt3a for example appears overall normal at birth (Li et al., 1992; Okano et al., 1999), but mice die 4 weeks after birth due to multiple developmental defects (Okano et al., 1999). It has been suggested that this is in part due to a disturbed neurogenesis in the SEZ of the forebrain and the hippocampal dentate gyrus, as NSCs loose DNA methylation on the gene bodies of neuronal genes and fail to activate those during differentiation (Wu et al., 2010). While defects in adult neurogenesis are unlikely to cause death of the entire organism, these data did reveal a key role of DNA-methylation in NSC differentiation with a clear decrease in postnatal neurogenesis. The authors also suggest that this was due to an increase in gliogenesis and hence a fate switch, but this is less clear as postnatal and adult NSCs also express astroglial markers, such as GFAP and some level of S100b (Beckervordersandforth et al., 2010), making it impossible to decide whether the increased cell population are NSCs or astrocytes. Conditional deletions of Dnmt3a in the developing nervous system (Nes1-Cre) have been reported to have a shortened lifespan as well, which has been attributed to postnatal motor neuron loss (Nguyen et al., 2007). Mouse embryos lacking Dnmt3b exhibit multiple developmental abnormalities, including rostral neural tube defects, and are not delivered to term (Okano et al., 1999). Thus, normal neural development is (at least partially) dependent on the presence of both *de novo* methyltransferases. Although full knockouts of Tet1 have been reported to be born overall normal (Dawlaty et al., 2011), recently new mutant alleles have been generated that are lethal during embryogenesis when outbred, at least partially due to “deformities in forebrain development associated with incomplete closure of the anterior neuropore” (Khoueiry et al., 2017).

Dnmt1 full knockout embryos have strong phenotypes and are early embryonic lethal (Li et al., 1992), while conditional deletions show a remarkably specific effect. Depletion of this methyltransferase in postmitotic neurons, using the CamK-Cre line, neither affected DNA methylation levels significantly, nor influenced postnatal survival of the animals, raising questions, which role Dnmt1 expression might play in postmitotic cells (Fan et al., 2001). Deletion of Dnmt1 in neural progenitors during development results in animal death (hours after birth in animals with high recombination rates; and significant neuronal loss in animals with reduced Cre activity) (Fan et al., 2001). Although after deletion of DNMT1 in the developing CNS up-regulation of some glial genes, like GFAP, have been observed, this occurred only at the end of neurogenesis and hence onset of gliogenesis *in vivo*, despite much earlier loss of DNMT1 using the Nestin-Cre line (Fan et al., 2005). Importantly, genomewide expression analysis should reveal best whether true fate changes occur—nowadays ideally done at single cell level. However, RNA-seq data do not reveal any indication for a fate switch when done early (E15 cortex Emx1^Cre^/Uhrf1, Ramesh et al., 2016) and highlight rather neuronal death as the main phenotypic consequence of hypomethylation in the brain and the GFAP increase as an indication of gliosis due to postnatal neuronal cell death when done later (Hutnick et al., 2009; Ramesh et al., 2016). Cortical degeneration appears not to be a consequence of altered fates, but rather due to another key role of DNA methylation: to stably silence repetitive elements [for Dnmt1 in particular ERVs like the intracisternal A-particle retroviruses (IAPs); (Walsh et al., 1998; Hutnick et al., 2009)]. Conditional deletions of the Dnmt1 partner Uhrf1 during cortical neurogenesis confirmed these findings and showed that despite profound demethylation primarily IAPs were de-repressed and that this is accompanied by postnatal neuronal degeneration (Ramesh et al., 2016). Interestingly, IAPs were up-regulated already at E12, yet neuronal death occurred only after the first postnatal week when neurons become functionally active. Indeed, many genes encoding for proteins involved in neuronal activity were dysregulated supporting again a role of DNA methylation in regulating neronal differentiation processes. Notably, despite loss of at least 25% of global DNA methylation no ectopic fates such as premature gliogenesis were observed in these mutants. Moreover the data indicated, that it is not the loss of DNA methylation, but rather the gain of 5hmC, which results in IAP activation during brain development, since the process can be rescued by simultaneous reduction of Tet2 and Tet3 (Ramesh et al., 2016). Thus, depleting key enzymes for DNA methylation maintenance or removal throws the epigenome out of balance, resulting in rather specific consequences for neuronal maturation and survival (Figure 4D). Similar to the phenotypes observed in brain development, deletion of DNMT1 in the retina and in oligodendrocyte progenitor cells show profound defects in the final maturation of photoreceptors and oligodendrocytes, respectively, but no generation of alternative fates (Table 1).

Development continues to some extent also in the adult brain, both in adult neurogenesis but also in the ongoing synaptic plasticity that constantly re-forms new synaptic connections. DNA modifications have also accredited functional roles in these processes including information storage and providing (in adult NSC niches) new mature neurons (Ninkovic and Götz, 2013). In the late 60s, an open debate was started, how neurons would be able store memory information for life, while the stability of the molecular building blocks of these cells is many orders of magnitudes shorter. Interestingly, DNA modifications, due to their mode of inheritance, have been frequently suggested as prime candidates for memory storage (Griffith and Mahler, 1969; Crick, 1984). Already in 1969 J.S. Griffith suggested “that the physical basis of memory could lie in the enzymatic modification of the DNA of nerve cells. It might be worth looking to see if there are unusual bases specific to nerve cell DNA, but in the absence of evidence to that effect, a plausible suggestion would be that the modification consists of methylation (or demethylation)” (Griffith and Mahler, 1969). During the last decades this concept has been regularly revived (Meagher, 2014). Indeed we know now, that the brain is, compared to other organs, especially active in remodeling DNA methylation patterns and a prominent source of scarce DNA modifications. For example, non-CpG methylation is common in neurons in contrast to other differentiated cell types (Guo et al., 2014), its occurrence is highly linked to the neuronal expression of Dnmt3a, as knockdown of this *de novo* methyltransferase abolishes CpH methylation (but not CpG methylation, which is mainly dependent on Dnmt1) (Guo et al., 2014). However, maybe the most surprising results stem from genetically modified, overexpression or knockdown mouse models of writer, reader, and eraser proteins of DNA modifications, resulting either in phenotypes affecting memory formation or consolidation [Tet1 (Kaas et al., 2013; Rudenko et al., 2013; Zhang et al., 2013), Dnmt1, Dnmt3a, and Dnmt3a2 (Miller and Sweatt, 2007; Feng et al., 2010; Oliveira et al., 2012, 2016)], emotional or maternal behavior [Dnmt3a (LaPlant et al., 2010), Mbd2 (Hendrich et al., 2001)], LTP [Mbd1 (Zhao et al., 2003)], or adult neurogenesis in the hippocampus [GADD45b (Ma et al., 2009), Tet1 (Zhang et al., 2013), Mbd1 (Zhao et al., 2003), Mecp2 (Smrt et al., 2007)] indicating that neuronal maturation or specific neuronal functions in particular neuronal plasticity might indeed be dependent on normal availability of the DNA modification machinery.

### Human model systems of DNA modifications and brain diseases

Interestingly, several neurodevelopmental disorders have also been linked to proteins involved in the regulation of DNA modification emphasizing their relevance in cerebral cortex development. Rett syndrome, a rare X-linked postnatal neurological disorder, was the first among this group, when it was discovered in 1999 that it is caused by mutations in the DNA methylation binding protein MeCP2 (Amir et al., 1999). In the meantime many more diseases have been added: for example, the *immunodeficiency, centromeric region instability, and facial anomalies syndrome* (ICF), caused by mutations in DNMT3B is often associated with mild cognitive and neurologic defects (Hagleitner et al., 2008). Similarly, childhood overgrowth syndrome, a developmental disorder resulting (amongst other phenotypes) in intellectual disabilities, is caused by mutations of the DNMT3A gene (Tatton-Brown et al., 2014). Moreover, it has been recently shown that brain tumors, use stem cell factors to interfere with astrocyte differentiation and the DNA methylation machinery (Bulstrode et al., 2017). Indeed some brain tumors are even driven by mutations in the isocitrate dehydrogenase 1 (IDH1). Mutations of this enzyme result in tumor cells which contain severely elevated global levels of DNA methylation. The reason for this is the abnormal accumulation of 2-hydroxyglutarate (2-HG), a powerful inhibitor of TET activity (Turcan et al., 2012). Thus, DNA methylation clearly also affects human NSC differentiation even though much more needs to be learnt about the exact mechanisms.

## Outlook

Taken together, the above mentioned experimental tests on the role of DNA methylation in cerebral cortex development do not lend much support to the model that it serves to repress alternative fates (Table 2). Besides GFAP up-regulation (Kim et al., 2016) there is not much evidence for aberrant glial fate instruction, including in genome-wide expression analysis (Hutnick et al., 2009; Ramesh et al., 2016), and no ectopic fate choices have been observed in any of the above mutants. Rather, a common theme is cell death, due to either the failure to fully differentiate and/or to repress repetitive elements (Ramesh et al., 2016), quite similar to what had been described in the postnatal retina (Rhee et al., 2012). Thus, the hypothesis that DNA methylation represses alternative fates has to be questioned, while the role in differentiation receives more support. Indeed, in the few studies of mouse mutants that examined the transcriptome, many aspects of immature cells, such as cell proliferation, fail to be repressed at later stages along with the failure to up-regulate genes involved in synaptic maturation. According to Wu et al., Prc2 mediated mechanisms could be involved in these processes as they showed that Dnmt3a-mediated DNA methylation adjacent to H3K4me3 high promoters interferes with Prc2 binding and H3K27me3 and thereby mediates up-regulation of neuronal progenitor genes (Wu et al., 2010). In addition or alternatively, Tet-mediated roles could be involved as described above from the Uhrf1 study (Ramesh et al., 2016). However, much remains to be understood about the repressive function of DNA-methylation in regard to differentiation and neuronal maturation. This is particularly evident from the poor correlation between changes in DNA-methylation and transcription. Further follow-up studies on the transcriptional changes that are crucial for the phenotypes aiming to correlate these to epigenetic mechanisms will hold the key to better mechanistic understanding of the mouse mutant phenotypes.

Indeed, so far virtually none of these phenotypes have been linked with precise sites in the genome being de-methylated, but always groups of sites, regions, or genes. This can be confounding as for example many methylation marks might have opposing roles in the body, such as maternal and paternal imprints that, respectively, reduce or activate growth (Barlow and Bartolomei, 2014). Thus, to elucidate which roles the epigenome plays in the brain, first we have to differentiate essential from specific and both from bystander marks, dissecting thereby secondary from causal DNA modifications. This can now be done by new options, which allow manipulating individual DNA modifications to evaluate their immediate causal effect on transcription and cell behavior. This new experimental field, collectively termed epigenome editing (Stricker et al., 2017), promises to deliver a better understanding of the role DNA modifications play during cortex development. Epigenome editing is mainly based on modified versions of the bacterial CRISPR system, allowing to precisely target any genomic locus in any cell. Fusing DNA modifying enzymes to dCas9 (the nuclease deficient targeting protein) has been proven to locally set or remove DNA modifications. So far, Tet1 and Dnmt3a have been used most prominently to show that DNA methylation on the accurate locus can indeed influence transcription of a gene close by (Amabile et al., 2016; Choudhury et al., 2016; Liu et al., 2016; Xu et al., 2016). However, we are still far from a comprehensive view about gene regulation by DNA modifications, especially during brain development, but studies successfully manipulating histone marks indicate this will be promising approach to study neurogenesis (Albert et al., 2017). Thus, tools of epigenetic engineering allowing methylating or demethylating specific genomic sites to investigate their function directly will help to causally link methylation of specific genes with functional phenotypes. This aim is more relevant than ever, as epigenome wide association studies (EWAS) suggest new targets for a variety of diseases on a regular basis (Stricker et al., 2017).

## Conclusion

While much remains to be done, experimental tests propose already a revision of the concept that DNA methylation would repress alternative fates (Tables 1, 2). Rather DNA methylation appears generally required for repression of ERVs, even though with striking cell type specificity. A further general concept that emerged is its role in orchestrating cell differentiation, but within a given lineage (neurons, oligodendrocyte progenitors, Table 2). The involvement of splicing as effector of changes in DNA methylation is an exciting new angle to pursue with more precise epigenetic engineering tools. Distinguishing essential from specific, and causal from secondary marks will be essential for neuro-epigenetics. New approaches promise to answer long outstanding questions and will likely facilitate the discovery that DNA modifications might have new unexpected roles in the brain.

## Author contributions

All authors listed have made a substantial, direct and intellectual contribution to the work, and approved it for publication.

### Conflict of interest statement

The authors declare that the research was conducted in the absence of any commercial or financial relationships that could be construed as a potential conflict of interest.
